# Genetic History of Hepatitis C Virus in Venezuela: High Diversity and Long Time of Evolution of HCV Genotype 2

**DOI:** 10.1371/journal.pone.0014315

**Published:** 2010-12-13

**Authors:** Maria Z. Sulbarán, Federico A. Di Lello, Yoneira Sulbarán, Clarisa Cosson, Carmen L. Loureiro, Héctor R. Rangel, Jean F. Cantaloube, Rodolfo H. Campos, Gonzalo Moratorio, Juan Cristina, Flor H. Pujol

**Affiliations:** 1 Laboratorio de Virología Molecular, CMBC, IVIC, Caracas, Venezuela; 2 Cátedra de Virología de la Facultad de Farmacia y Bioquímica, Universidad de Buenos Aires, Buenos Aires, Argentina; 3 Unité Emergence et Co-évolution virale, Etablissement Français du Sang Alpes-Méditerranée, Marseille, France; 4 Departamento de Técnicas Nucleares Aplicadas, Facultad de Ciencias, Centro de Investigaciones Nucleares, Universidad de la República, Montevideo, Uruguay; 5 Unidad de Biofísica de Proteínas, Institut Pasteur de Montevideo, Montevideo, Uruguay; University of Pretoria, South Africa

## Abstract

**Background:**

The subtype diversity of the hepatitis C virus (HCV) genotypes is unknown in Venezuela.

**Methodology/Principal Findings:**

Partial sequencing of the NS5B region was performed in 310 isolates circulating in patients from 1995 to 2007. In the samples collected between 2005 and 2007, HCV genotype 1 (G1) was the most common genotype (63%), composed as expected of mainly G1a and G1b. G2 was the second most common genotype (33%), being G2a almost absent and G2j the most frequent subtype. Sequence analysis of the core region confirmed the subtype assignment performed within the NS5b region in 63 isolates. The complete genome sequence of G2j was obtained. G2j has been described in France, Canada and Burkina Fasso, but it was not found in Martinique, where several subtypes of G2 circulate in the general population. Bayesian coalescence analysis indicated a most recent common ancestor (MRCA) of G2j around 1785, before the introduction of G1b (1869) and G1a (1922). While HCV G1a and G1b experienced a growth reduction since 1990, coincident with the time when blood testing was implemented in Venezuela, HCV G2j did not seem to reach growth equilibrium during this period.

**Conclusions/Significance:**

Assuming the introduction of G2j from Africa during the slave trade, the high frequency of G2j found in Venezuela could suggest: 1- the introduction of African ethnic groups different from the ones introduced to Martinique or 2- the occurrence of a founder effect. This study represents an in-depth analysis of the subtype diversity of HCV in Venezuela, which is still unexplored in the Americas and deserves further studies.

## Introduction

Around 170 million persons in the world are thought to be infected with hepatitis C virus (HCV). The highest rates of HCV infection are found in some North African countries like Egypt, and in the Western Pacific regions [1]. Information is scarce about the epidemiology of hepatitis C virus (HCV) in South America. Overall prevalence of HCV antibodies in non-Amerindian populations from South America is around 1% [2]–[4]. In the Americas, a gradient of exposure to HCV infection has been found from rural to urban populations groups. The prevalence of this viral infection correlates to parenteral risk of exposure, including iatrogenic and intravenous drug abuse [5], [6].

Six HCV genotypes, and a large number of subtypes in each genotype, have been described. HCV genotypes 1, 2, and 3 have a worldwide distribution. HCV subtypes 1a (G1a) and G1b are the most common genotypes in the US and are also predominant in Europe. Although HCV G2a and G2b are relatively common in America, Europe, and Japan, subtype 2c is found commonly in northern Italy. HCV G3a is frequent in intravenous drug abusers in Europe and the United States. HCV G4 appears to be prevalent in Africa and the Middle East, and G5 and G6 seem to be confined to South Africa and Asia, respectively [7], [8]. These last genotypes are also increasingly found in other continents, frequently associated to particular transmission routes or epidemiological settings [9].

The low prevalence of HCV infection in many native population groups from Latin America strongly suggests that HCV is not autochthonous to these regions. This situation is in contrast with the high prevalence rates and genetic diversity found for this virus in Africa and South Eastern Asia, which might be associated to the origin of this virus [8]. No autochthonous HCV genotype has been described in the region, except for some South American lineages of G1 [10] which probably emerged during their evolution in the New World. Changes in hepatitis C virus (HCV) genotype distribution with time have been reported in several countries. In Venezuela, a significant reduction of the circulation of HCV G1b was observed in the last decade, with the increase of circulation of G2 [11].

It is known that the subtype diversity is only fairly appreciated by studying the 5′- non-coding (5′NC) region of the HCV genome, and that the genetic analysis of a small fragment of the NS5B region does generally reproduce the genotypic variability found in the complete viral genome [12], [13]. The subtypic variability has been scarcely studied in Latin America. Cristina et al. [14] found a relatively low intragenotypic diversity of HCV genotype 1 in Latin America, compared to that observed in Africa. Martial et al. [15] found a great diversity of genotypes, and particularly of subtypes of G2, in the Caribbean island Martinique. This genotype is not very prevalent in other countries from Latin America [14], [16]–[19].

The aim of this study was to analyze the genetic diversity of HCV in Venezuela and to reconstruct the genetic history of the more prevalent epidemics subtypes. An unusual subtype of G2, G2j, was frequently found infecting Venezuelan patients. The complete coding region of the genome was analyzed and allowed to confirm the previous assignment of HCV G2j, which may have been be circulating since 1785 in Venezuela.

## Results

### Amplification and sequencing of the NS5B region

A total of 479 sera, previously analyzed by sequencing of the 5′-NC region, were analyzed, 166 collected between 1995 and 2004 and 313 between 2005 and 2007. Using 3 nested PCR reactions with different combinations of primers (Table 1, see Materials and methods), NS5b region could be amplified and sequenced in 310 of them, 80/166 (48%) of samples from the first period and 230/313 (74%) from the latest one. The efficiency of NS5B amplification was evaluated among the samples from the second period of time, since these sera were not subjected to multiple freeze-thawing. HCV samples, assigned as G2b by analysis in the 5′-NC region, were less frequently amplified in the NS5B region. While 25/38 (66%) of G2b isolates could not be amplified by the different PCR methods adopted to amplify the NS5B region, only 59/275 (21%) of isolates belonging to other genotypes or subtypes could not be amplified (p<0.0001).

**Table 1:** Primers used for amplification and sequencing of the HCV genome.

Primer	Sequence	Nucleotides	Polarity
**939P^1^**	ctgtgaggaactactgtctt	45–64	Sense
**940P^1^**	ttcacgcagaaagcgtctag	63–82	Sense
**209N^1^**	atactcgaggtgcacggtctacgagacct	321–349	Antisense
**211N^1^**	cactctcgagcaccctatcaggcagt	288–313	Antisense
**C4N**	accaattcatcatcatatcccaagccattcg	1290–1320	Antisense
**C5P**	gactgctagccgagtagtgttgggtcgcgaaag	245–277	Sense
**C6N**	cattggtcacctggtacaccccggacacgttg	926–957	Antisense
**C9N**	cattggtcaccatgtagctggtactggtgttc	925–956	Antisense
**C10N**	gagatgcattccccactttctcgcacgggacgcacccg	1013–1050	Antisense
**693P**	aaybtgggyarrgtcatcg	693–711	Sense
**1298P**	atggcntgggayatgatg	1293–1310	Sense
**1598P**	aatggcagntggcayathaa	1590–1609	Sense
**2001N**	tcatccangtgcanccgaacca	1986–2007	Antisense
**2001P**	tggttcggntgcacntggatga	1986–2007	Sense
**3008P**	tgttygacrtdaccaadtgg	3006–3024	Sense
**4175P**	atcaatcccaacatyagractgg	4161–4183	Sense
**4290P**	tcatmatatgcgaygartgcca	4276–4297	Sense
**5045P**	ggcctcacacayatagaygcyca	5031–5053	Sense
**5082P**	aggaagtgggcrtctat	5043–5059	Antisense
**5930P**	gtngchttyaarrtcatg	5931–5948	Sense
**5930N**	catgayyttraadgcnac	5931–5948	Antisense
**6100P**	tggatgaayaggctyatwgcctttgc	6084–6109	Sense
**6100N**	gcaaaggcwatragcctrttcatcca	6084–6109	Antisense
**6177N**	gactccgbcacgtagtg	6138–6154	Antisense
**6177P**	cactacgtgvcggagtc	6138–6154	Sense
**6522P**	ctggcargggacmtttccyatcaa	6506–6529	Sense
**6522N**	ctggcargggacmtttccyatcaa	6506–6529	Sense
**6623N**	gcgccaccytccadat	6596–6611	Antisense
**6712P**	gagttyttytcctgggtgga	6709–6728	Sense
**7071P**	gacatggtsgatgchaa	7025–7041	Sense
**7071N**	gacatggtsgatgchaa	7025–7041	Antisense
**7670P**	tgctccatgtcmtactcctggac	7663–7685	Sense
**7743N**	gtccangagtangacat	7605–7621	Antisense
**7743P**	atgtcntactcntggac	7605–7621	Sense
**8245N^1^**	tcraarcagcgggtatcatacgggatcccca	8245–8275	Antisense
**8245P^1^**	tggggatcccgtatgatacccgctgyttyga	8245–8275	Sense
**8276P^1^**	ctccacagtcactgagagcgacat	8276–8299	Sense
**PR3P**	tatgayacccgctgytttgactc	8256–8278	Sense
**hep101^1^**	atacccgctgctttgactc	8260–8278	Sense
**hep105^1^**	atacctagtcatagcctccgtga	8617–8639	Antisense
**hep102^1^**	agcatgatgttatcagctcc	8681–8700	Antisense
**8580N^1^**	ggcggaattcctggtcatagcctccgtgaa	8557–8580	Antisense
**8625N^1^**	gartacctrgtcatagc	8625–8641	Antisense
**8645N^1^**	ggcggaattcctggtcatagcctccgtgaa	8616–8645	Antisense
**9325N**	ctacrgtaagyaggagtaggc	9325–9345	Antisense

The numbers refer to the position in the sequence of a H77 G1a isolate, accession number in Genbank AF009606. Primers were obtained from the literature^1^ [53], [54], [63] or designed in this study.

### HCV subtype distribution in Venezuela

**Figure 1:**
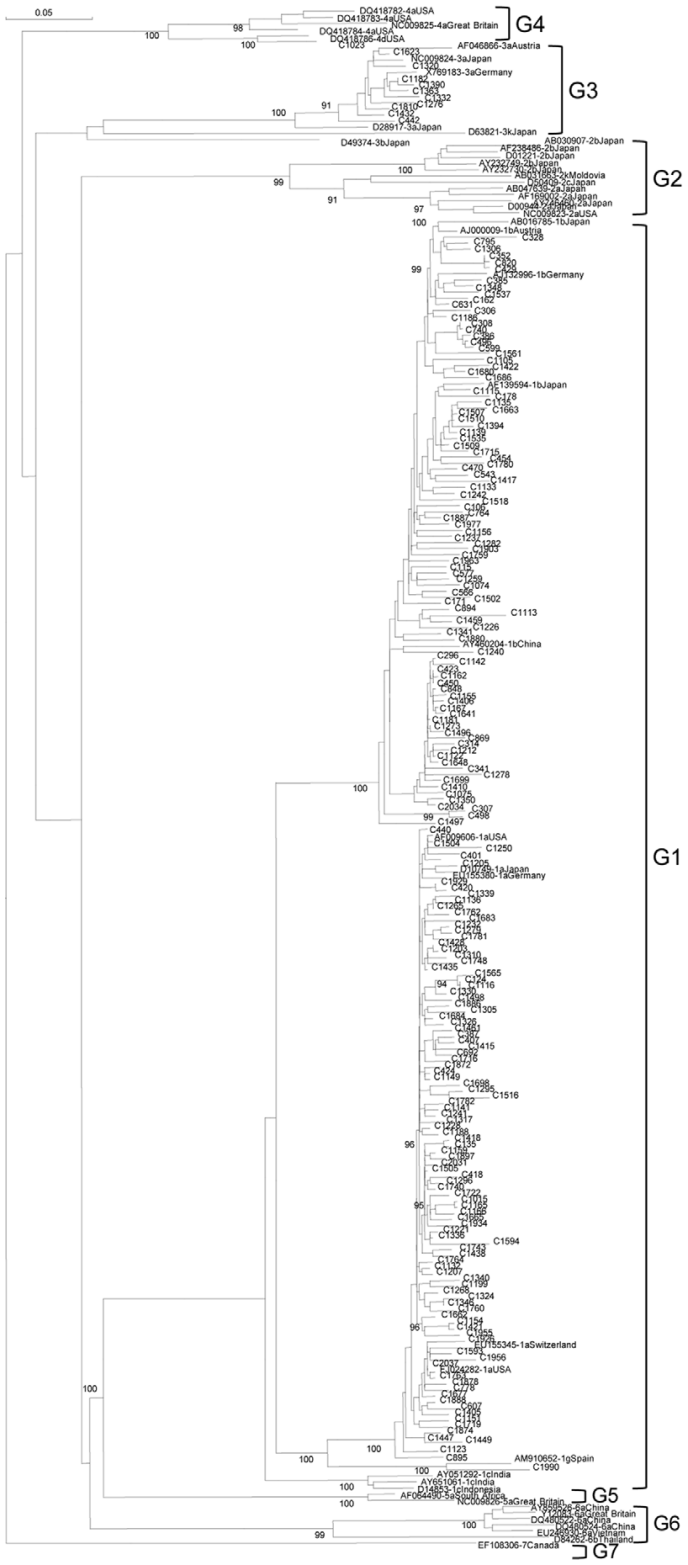
Phylogenetic analysis of non G2 HCV Venezuelan strains. NS5B region (256 nt, 8302–8556). Neighbor Joining method. Isolates are designated by their GenBank accession number, followed by their subtype assignment and country of origin. Venezuelan isolates, are numbered and preceded by a C. Bootstrap values over 90% are shown in the tree.

**Figure 2:**
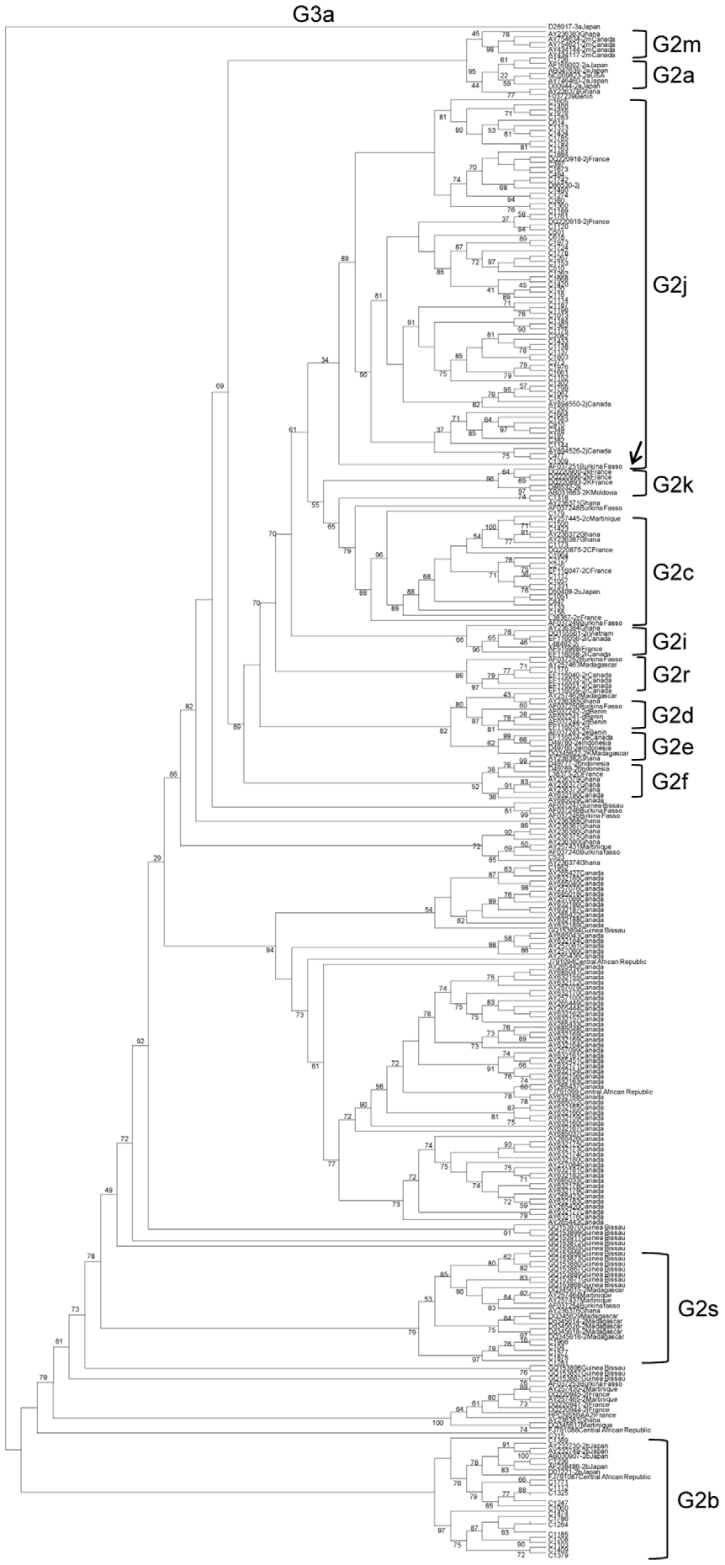
Phylogenetic analysis of G2 HCV Venezuelan strains. NS5B region (209 nt, 8343–8549). Maximum likelihood analysis. Isolates are designated by their GenBank accession number, followed by their subtype assignment and country of origin. Venezuelan isolates, are numbered and preceded by a C. Bootstrap values over 90% are shown in the tree. The arrow indicates the strain from Burkina Fasso (GenBank accession number AF037251) which appears as an ancestor of the G2j strains.

The present distribution of HCV subtypes in Venezuela was evaluated in the samples collected between 2005 and 2007. HCV G1 was composed as expected of mainly G1a and G1b, and one strain belonged to G1g (Figure 1). G4 and G3 isolates belonged to subtype d and a, respectively (Figure 1). Unexpectedly, G2a was almost absent from Venezuelan isolates. The specimens classified as G2a according to the analysis of the 5′NC region, were indeed mostly G2j, a subtype found previously only in Canada and France. This subtype was very prevalent in Venezuela during the 13 years analyzed (Figure 2). Based on the analysis of the NS5B region, and even with the bias due to the low rate of amplification of genotype 2b isolates, the most frequent subtypes in Venezuela were 1a, 1b and 2j, with around 37, 26 and 21% prevalence respectively. Several other subtypes and putative subtypes of G2 were also identified (Table 2). The gender prevalence among the HCV infected patients was 58% of males and no significant difference was found in the gender prevalence between the 3 most common subtypes. There was no significant difference in the mean age of the 74 patients whose sera was analyzed between 2005 and 2007, mean age was 41 years for HCV G1a as well as G1b and 42 years for G2j.

**Table 2:** HCV genotypes and subtypes distribution in Venezuela during 2005–2007.

Subtype assigned by NS5b region	N (% over a total 230)(Isolates)	Genotype distribution
**1a**	84(37%)	144 (63%)
**1b**	59(26%)	
**1g**	1(0.4%)(C1990)	
**2j**	48(21%)	77 (33%)
**2b**	14(6%)	
**2c**	7(3%)	
**2a**	1(0.4%)(C1726)	
**2r**	1(0.4%)(C1170)	
**2s^1^**	4(2%)(C104,C1281,C1577,C1875,C1966, others)^2^	
**2^3^**	2(0.9%)(C523,C1318,C1952)	
**3a**	9(4%)	9 (4%)

1: New subtype assignment proposed based on the analysis of core, E1–E2 and NS5B genomic regions. 2: C104 sample from the 1995–2004 period not included for prevalence determination. Others: G2s strains from Guinea-Bissau, Burkina Fasso, Ghana, Madagascar, and Martinique. 3: Unclassified G2 subtypes. C523 sample from the 1995–2004 period not included for prevalence determination.

The diversity of subtypes in G2 was confirmed by phylogenetic analysis by Neighbor Joining method (data not shown) and Maximum Likelihood inference (Figure 2). A total of 130 sequences of isolates from Africa, and 6 from Martinique, available at GenBank, were included in the phylogenetic analysis. The G2j clade was composed of 65 sequences from Venezuela, 4 from Canada and 5 from France (two of each shown in the tree). In addition, a strain from Burkina Fasso (AF037251) was grouped as an ancestor of the G2j strains (Figure 2).

**Figure 3:**
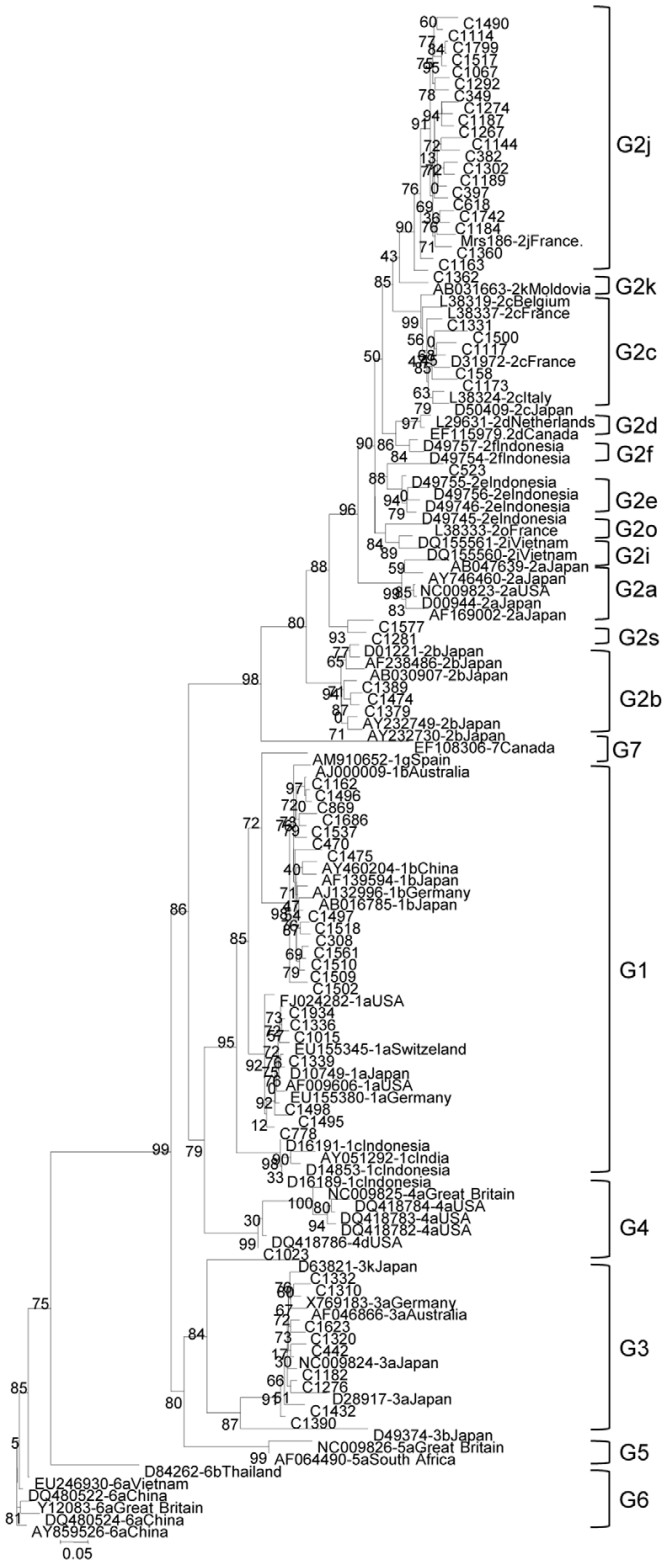
Phylogenetic analysis of HCV Venezuelan strains in the Core region (507 nt, 372–877). Maximum likelihood analysis. Isolates are designated by their GenBank accession number, followed by their subtype assignment and country of origin. Venezuelan isolates, are numbered and preceded by a C. Bootstrap values over 90% are shown in the tree.

In order to support the genotype assignment based on the NS5B region, sequence analysis was performed on the core region for selected isolates, confirming the subtype assignment performed within the NS5B region in 63 of them (Figure 3). In addition, the core sequence from a French G2j isolate grouped with the Venezuelan sequences. In the core region, the G2j isolates displayed an intra-subtype mean nucleotide identity of 91%, 90% with G2k and G2c sequences, the closest available from the GenBank. A 100% concordance was found between the subtype assignment in the NS5B and core region, implying no evidence of recombination in the samples analyzed in these regions. A new subtype G2s, proposed for 5 Venezuelan isolates (C104, C1281, C1577, C1875 y C1966), is supported by the phylogenetic analysis of three HCV genomic regions: NS5B (Figure 2), core (Figure 3) and E1–E2 region (this last analysis not shown). In addition, isolates C523, C1318 and C1952 may belong to unassigned subtypes of HCV G2 (Table 2).

### Complete genome analysis of G2j isolates

**Figure 4:**
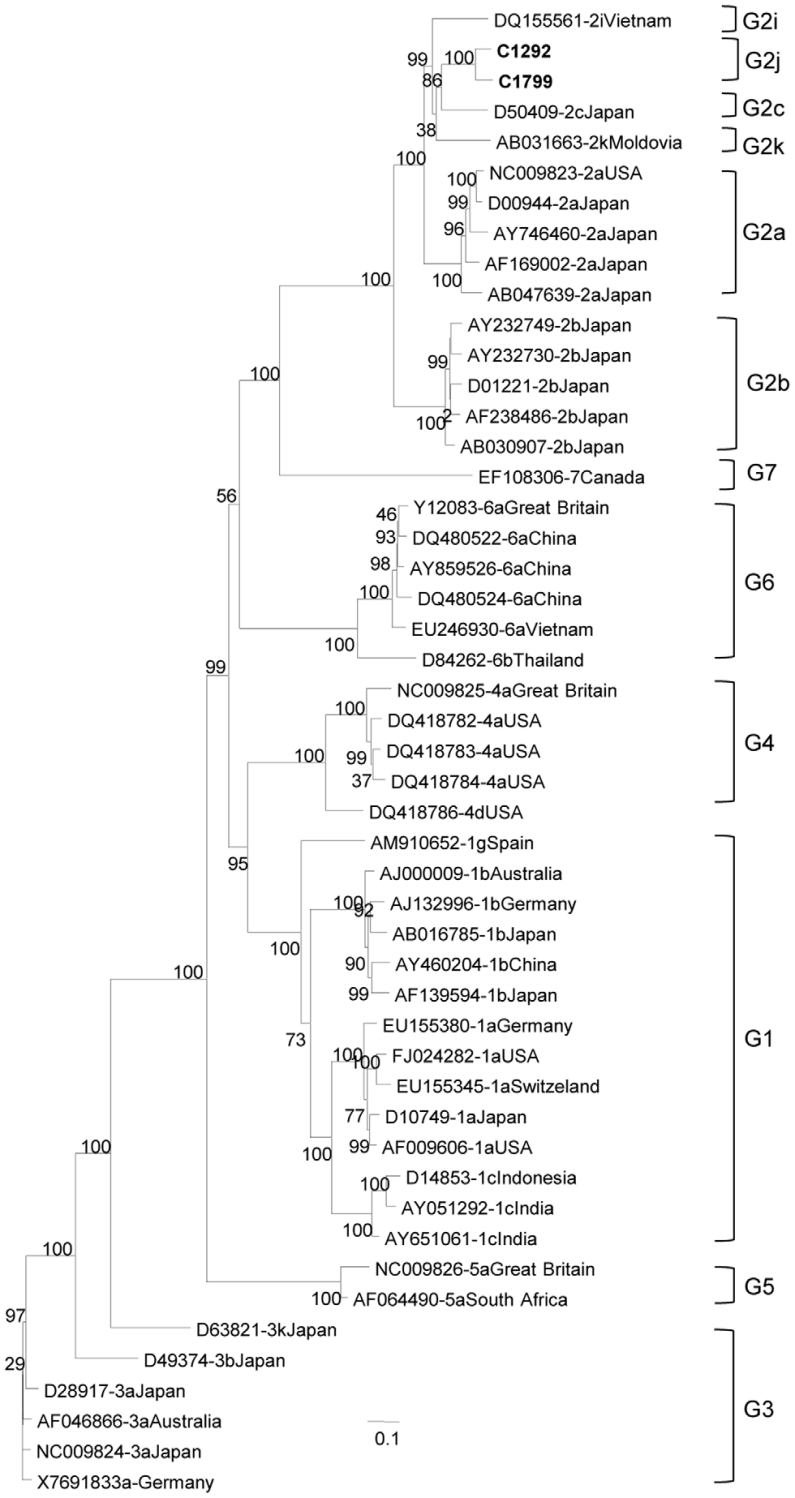
Phylogenetic analysis of G2j HCV Venezuelan complete genome strains. Almost complete genome (9046 nt, 82–9127). Maximum likelihood analysis. Reference isolates are designated by their GenBank accession number, followed by their subtype assignment and country of origin. Venezuelan isolates are shown in bold. Bootstrap values over 90% are shown in the tree.

The first complete genomic sequence of one G2j isolate (C1292, nt 82 to 9347, according to reference strain AF009606) and almost complete sequence of another isolate (C1799, nt 82 to 9127, according to reference strain AF009606) were obtained, by sequencing 10 overlapping PCR-amplified fragments (Table 1). The G2j Venezuelan strains clustered together, separately from the other G2 sequences (Figure 4). Eventual genomic recombination events of G2j sequences with other genotypes or subtype 2 genomes were ruled out by Simplot analysis (data not shown). The complete coding region of G2j isolate C1292 exhibited less than 88% amino acid identity with G2c and G2k isolates, less than 87% with G2a ones, and 86% and 85% with G2i and G2b isolates respectively.

**Figure 5:**
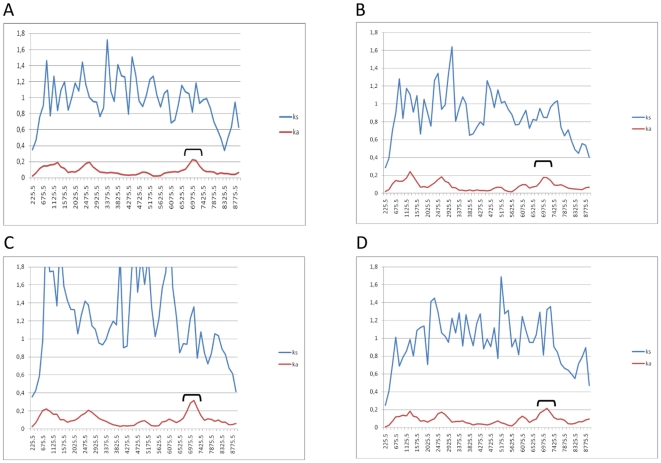
Profiles of synonymous and non synonymous distances between Venezuelan G2j strain C1292 and HCV strains from other G2 subtypes along the HCV polyprotein(9036 nt, 342–9374). Synonymous substitutions are shown by blue line (Ks) and non-synonymous substitutions by a red line (Ka). Comparison of sequences from C1292 strain and strains D00944 (G2a) (A); D10988 (G2b) (B); D50409 (G2c) (C) and AB032663 (G2k) (D). The x-axis depicts the coding nucleotide position number in the middle of the window, and the y-axis depicts distance. NS5A region is indicated by a bracket.

To observe the degree of genetic variation along the coding region of the HCV genome of Venezuelan G2j, we compared the similarity index along the HCV polyprotein among strain C1292 and reference strains from major genotype 2 subtypes. A significant degree of genetic variation can be seen among C1292 (G2j) and strains belonging to other G2 subtypes (Figure 5). This is particularly significant in specific regions like core, E1 and NS5A (Figure 5).

To study the variation in the rates of synonymous and non synonymous substitutions within the C1292 polyprotein, this strain was compared with strains from different types and subtypes. Synonymous distances are significantly higher than non synonymous ones for all pair wise comparisons (Figure 5). For that reason, the ratio of non synonymous distance/synonymous distance (*k_a_/k_s_*) is low along the whole sequence. This has usually been associated with purifying selection acting at the level of amino acid conservation. Although low non synonymous rates were found throughout the polyprotein, it is possible to observe in all comparisons an increase of non synonymous distance in the NS5A region of the genome (Figure 5), suggesting that NS5A region of G2j strain C1292 may have a number of non synonymous substitutions by comparison with other G2 subtype strains.

The NS2/NS3 cleavage sites [20] were conserved in the two G2j isolates. The amino acids motifs surrounding these sites were essentially similar to the ones present in the G2c isolate, except for the presence of an Asparagine instead of an Serine in the N terminal of the NS4a of C1799 isolate, although this substitution is also observed in an G3b isolate [20] (data not shown). There was a strong conservation of potential N-linked glycosylation sites in the envelope protein as other genotypes. The E1 protein contained four sites (positions 196, 209, 234 and 305; numbered as in strain D10988) which are conserved among all variants. Glycosylation in the E2 protein was more variable. The sequence of G2j predicted ten sites (positions 417, 423, 430, 448, 534, 542, 558, 578, 627 and 649) conserved amongst C1292 and C1799 strains. An eleventh site was found at position 477 in C1799 strain.

**Figure 6:**
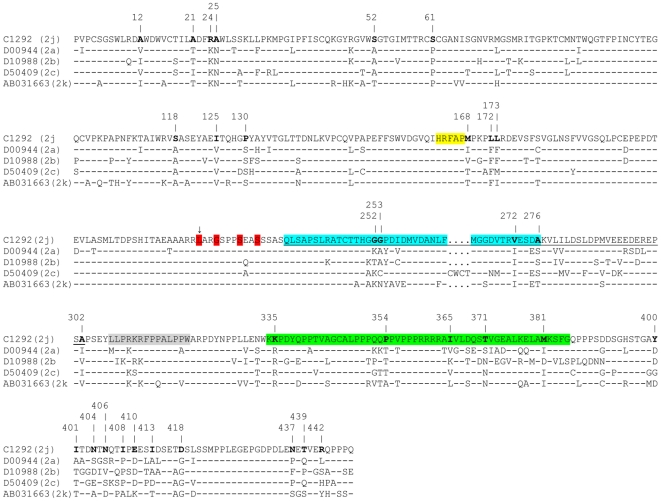
Alignment of complete NS5A amino acid sequences from Venezuelan G2j strain C1292 and G2 reference strains (1338 nt, 6258–7595). Strains are indicated by accession numbers for the reference strains and their subtype is indicated between parentheses. Amino acid sequences are indicated by the one-letter code. Identity to C1292 strain is indicated by a dash. The interferon-sensitivity determining region (ISDR) is indicated in blue, the V3 region is indicated in green. NS5A region interacting with NS4A is shown in yellow. The region interacting with PKR is shown underlined. Major phosphorilation site is shown in red and indicated by an arrow, serine residues required for hyperphosphorilation are shown in red. Other regions suggested to be under selective pressure during therapy are shown in grey. Substitutions found in C1292 are shown in bold and their position is indicated by numbers at the top of the alignment.

Due to the fact that NS5A is a critical component of HCV replication and is involved in several cellular processes, such as interferon resistance [21] and apoptotic regulation [22], NS5A sequences from G2j strain C1292 was aligned with corresponding sequences of reference strains from other G2 subtypes, in order to observe the amino acid changes in C1292. Interestingly, amino acid substitutions were observed in all domains of NS5A of G2j strain C1292 (Figure 6). Unique amino acid substitutions were found in the interferon-sensitive determining region (ISDR) and the Protein Kinase R (PKR) interaction region of this protein by comparison with other genotype 2 sub-types (Figure 6).

**Figure 7:**
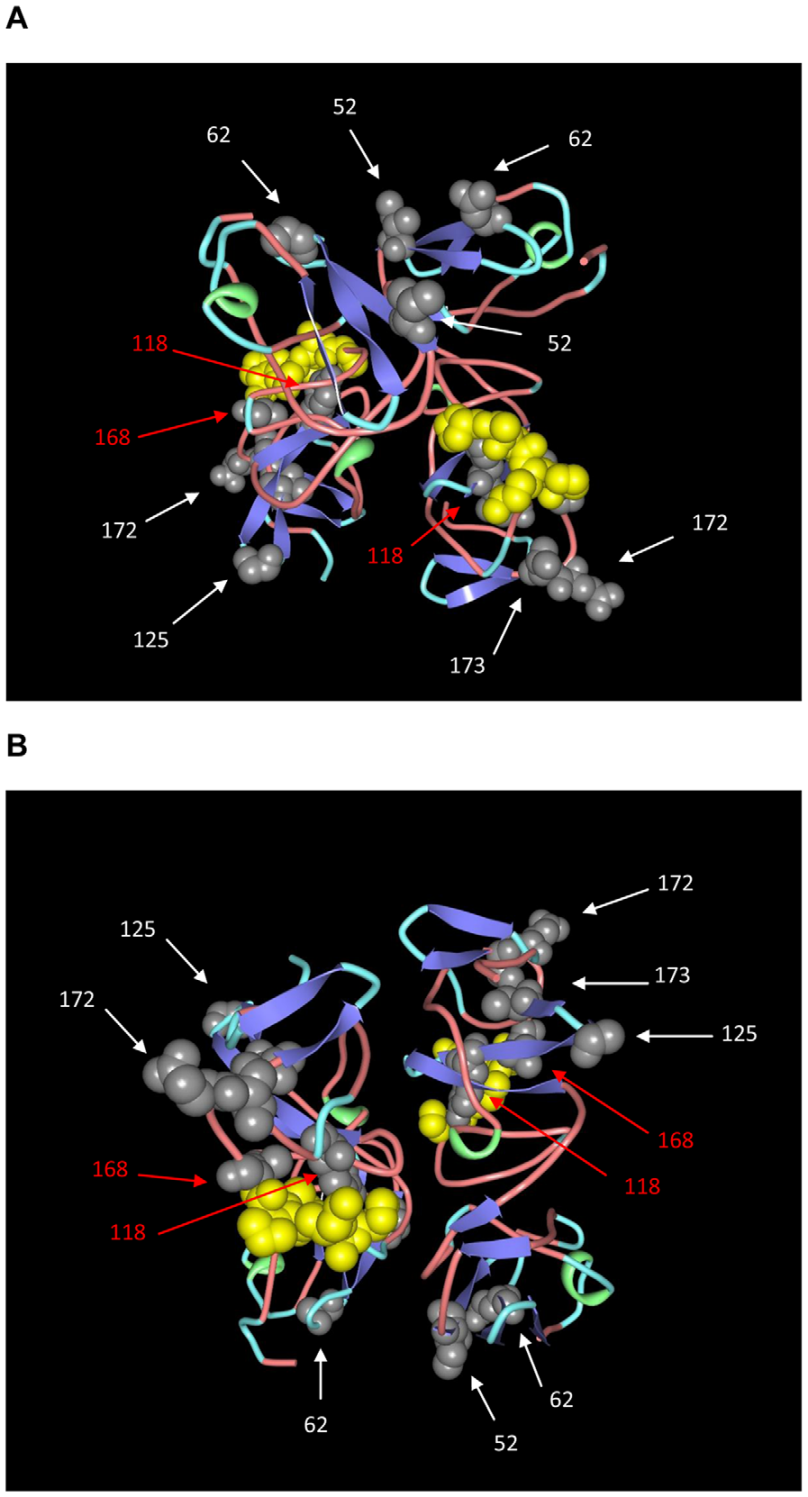
3D model of domain I of NS5A protein. A dimeric model of domain I NS5A protein (PDB accession number 3FQQ) is shown. The molecules are colored according to conformational type (turn is shown in light blue, coil in light red, helix in green and strand in blue). Amino acid positions corresponding to NS4A interactions are shown in space filling representation in yellow. Amino acid substitution positions found in C1292 (G2j) are shown in space filling representation in grey and their positions are indicated by numbers and shown by arrows. Red arrows indicate residues spatially close to those known to interact with NS4A protein. Two views of the molecules, rotated under the *x*-axis are shown in (A) and (B), respectively.

The crystal structure of a dimeric form of domain I of NS5A (residues 33 to 202) has been recently resolved [23]. The amino acid substitutions found in domain I of NS5A of strain C1292 isolated in Venezuela were mapped spatially in the 3D structure of this domain of NS5A protein (Figure 7) (Protein Data Bank accession number 3fqq) [23]. Several substitutions occur throughout domain I of NS5A protein. Interestingly, two substitutions at position 118 and 168 of NS5A protein map spatially close to residues known to interact with NS4A protein (Figure 7).

### Epidemic history of the most common subtypes of HCV circulating in Venezuela

**Figure 8:**
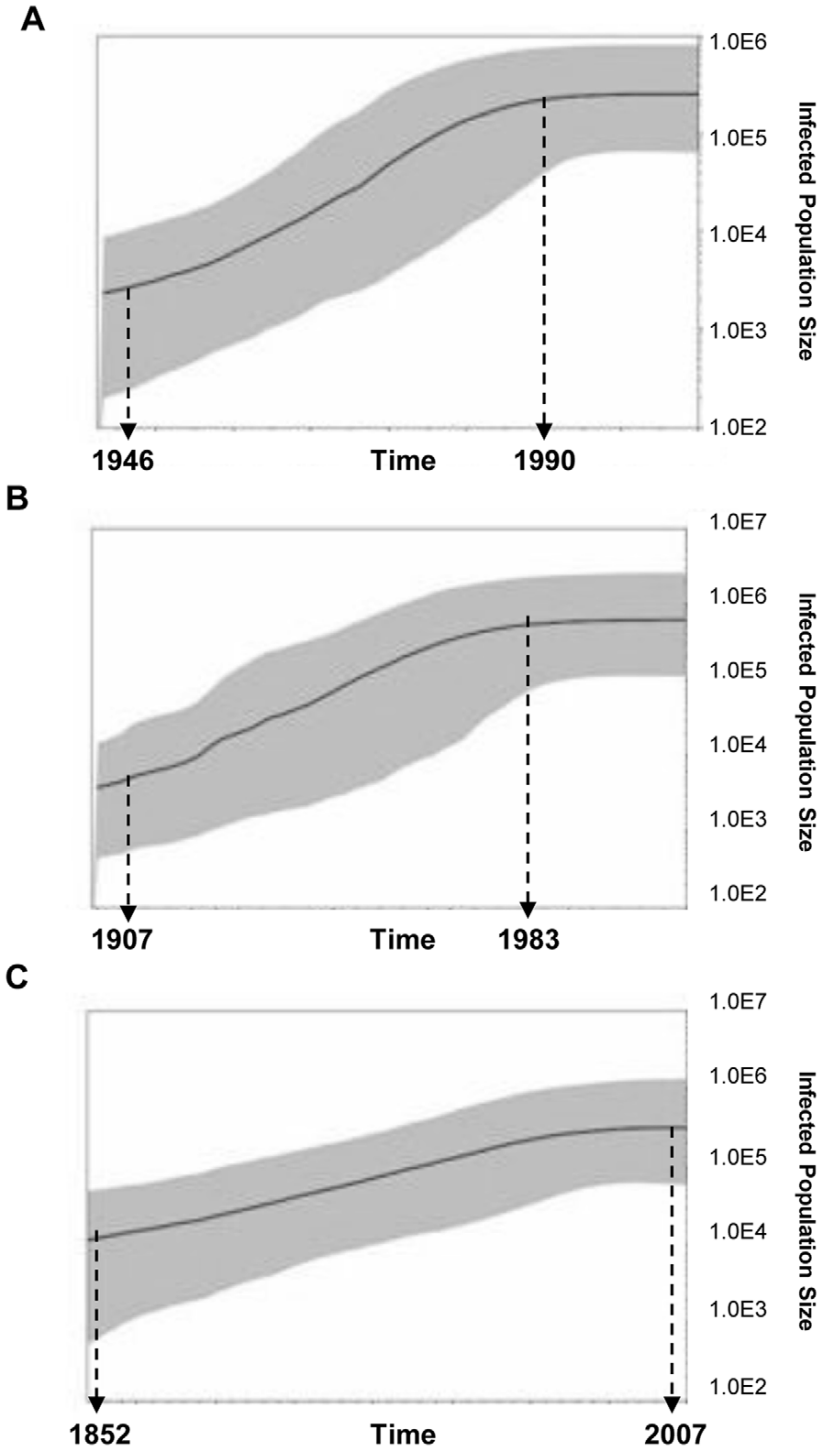
Bayesian Skyline plot analysis of Venezuelan HCV isolates. G1a (A), G1b (B) and G2j (C) isolates. Solid line represents the estimation of effective size number and shaded area the 95% confidence limit of HPD (High Population Density).

Bayesian coalescence analysis was performed on G1a, G1b and G2j NS5B sequences from Venezuela. For each demographic and the molecular clock models a chain lengths of 170 million were used and sampled every 1000 states. For the 3 subtypes analyzed, the best fitted model was the exponential growth with relaxed molecular clock not correlated exponential. These results suggest an exponential increase and dissemination of the 3 HCV subtypes in Venezuela. The data for the 3 HCV subtypes was analyzed by using the Bayesian Skyline Plot (BSP) in order to describe the demographic history of these viral populations (Figure 8). HCV G1a and G1b exhibited a similar epidemic history, characterized by a logistic growth, with an initial phase of unknown duration and low population increase, followed by a well defined phase of exponential growth and finally an equilibrium phase. The epidemic history of G1b precedes the one of G1a by several decades. While the G1b population increased exponentially between 1911 and 1986, G1a population experienced an exponential growth between 1952 and 1990. In contrast, the G2j population did not reach growth equilibrium during this period.

The most recent common ancestor (MRCA) was evaluated by using a fixed substitution rate for NS5b region of 4.1×10^−4^ substitutions per site/month [24], which has proven to be reliable for the reconstruction of epidemic events [25]. In addition, the substitution rate determined from these data sets was fairly similar to the one reported by Pybus et al. [24] (data not shown). The use of a predetermined substitution rate allowed a more reliable determination of the demographic parameters. MRCA of G2j was evaluated around 1785 (Effective Sample Size, ESS 236), before the introduction of G1a (1922, ESS 243.8) around the same time than G1b (1869, ESS 152.1) (Table 3).

**Table 3:** Epidemic history of HCV subtypes 1a, 1b and 2j in Venezuela.

HCV subtype	Number of isolates	Substitution rate(pre-ajusted)	MRCA (HPD^1^ 95%)
NS5B 1a	94	5×10^−4^ s/site/yr	1922 (1902–1942)
NS5B 1b	90	5×10^−4^ s/site/yr	1869 (1834–1901)
NS5B 2j	62	5×10^−4^ s/site/yr	1785 (1727–1837)

1: HPD: Highest Posterior Density.

## Discussion

While HCV G1 is the most prevalent genotype throughout the Americas, the frequency of HCV G2 varies from country to country. Although it is not very common in Brazil, Canada, Chile, Mexico, or the USA, the HCV G2c genotype is the second most frequently isolated, after G1 [11], [14], [26]–[30], in Argentina [17]. In Venezuela the prevalence of HCV G2 has been increasing over the past decade [11]. In the present study HCV G2 was found to be more diverse than suspected, and more diverse than G1. A similarly high degree of of G2 diversity was previously described in Martinique, although the subtype distribution was different [15]. The HCV G2j subtype that predominates in Venezuela was not found in Martinique [15], although it has been described in France, Canada and Burkina Fasso [12], [31]–[33]. The classification of Venezuelan isolates as G2j was confirmed by phylogenetic analysis in the core and E1–E2 regions, and by sequencing the complete genome of one isolate. The complete and nearly complete genomic sequences of G2j showed that this subtype is different from others previously described, and has several features that distinguish it from the closest related subtypes, G2a, G2c and G2k. In addition, at least one new subtype of HCV G2, proposed as 2s, was found in Venezuela. This new designation was based on the phylogenetic analysis of at least three informative regions of the HCV genome (core, E1–E2 and NS5B), and the presence of the new subtype in six different isolates, 5 from Venezuela and one from Guinea-Bissau [33].

Venezuelan strains of HCV G2 could have been introduced into the Americas with the slave trade. Between the 15th and 19th centuries over 11 million Africans were forcibly removed from Africa, principally from coastal areas in West and West Central Africa, and shipped to the Americas [34]. While it is difficult to determine the origins of specific ethnic groups in each country [35] it is thought that most of the slave population in Venezuela was brought from West Africa, with a primary acclimation stay in the Caribbean islands, particularly the Dominican Republic (Castro de Guerra, D., personal communication). The HCV G2 diversity found in Venezuela is compatible with its having been brought from West Africa, where the genotype appears to have been circulating for a long time [33]. It is interesting that one G2r isolate, C1170, clustered together with G2r isolates infecting Canadian immigrants from Haiti and Dominican Republic [12], perhaps reflecting the stays of West African slaves on the island of Hispaniola.

HCV G2 sequences are now available from several West African countries, and their phylogenetic tree displays a spatial structure grouped in clusters corresponding to geographic areas: 1) Cameroon and Central African Republic, 2) Benin, Ghana and Burkina Fasso, 3) Gambia, Guinea, Guinea-Bissau and Senegal and 4) Madagascar [33]. The G2j sequences were grouped within the second cluster, and one G2j related isolate was found in Burkina Fasso. A possible origin of G2j in this former French colony is compatible with the finding of several G2j strains in Canada and France [12], [31], [36]. Although significantly less represented than the G2j subtype, other Venezuelan G2 isolates were related to the African clusters 1 and 3 (above), but none were related to the Madagascar cluster. This diversity of G2 subtypes suggests diverse geographic origins for the slaves brought to Venezuela. Population descendants from Congo, the Democratic Republic of Congo and Angola might have been frequent in Venezuela. Interestingly, G2 is less frequent than G4 in Congo and the Democratic Republic of Congo [37], [38]. No information is available for Angola. The puzzling question is how to explain the predominance of G2j in a context where more G2 diversity would be expected, but this could represent a founder effect [39] of G2j in Venezuela.

Bayesian coalescence analysis indicated an MRCA of G2j in Venezuela around 1785, very close to the MRCA of G2c in France (1791) [40]. This considerably predates the MRCA's for G1b in Venezuela (1869), which is similar to that in Brazil, Chile and US [41]–[43] but older than reported in Argentina and China (1964–1979) [44], [45], and G1a (1922), which is similar to that in Brazil and US [42], [45]. Interestingly, the epidemic history of G2j is also distinct from that of the G1 subtypes. While the G1 strains displayed an exponential growth through 1985–1990, before reaching a plateau coincident with the implementation of HCV blood bank testing in Venezuela in 1994 [11], the G2j strains do not seem to have reached growth equilibrium during this period.

Some of the HCV epidemics have been associated with particular transmission routes, such as intravenous drug use in the case of HCV G3a [46], and parenteral treatment of schistosomiasis with G4 in Egypt [47]. Sexual transmission of HCV seems to occur but is thought to be infrequent [48], [49]. A recent study, however, suggests that sexual transmission might have played an important role in the transmission of HCV G2 over the past centuries in Guinea Bissau, where this genotype is highly predominant [50]. Although there is no evidence that a particular variant of HCV might be more efficiently transmitted by a specific route, this issue has not been addressed adequately. It has been shown that HIV co-infection could be an important factor contributing to an efficient sexual transmission of HCV in African countries [51], [52]. It is not known if sexual transmission might play a role in the dissemination of G2j in Venezuela.

In conclusion, an unexpected HCV variant, G2j, was found at a relatively high frequency in Venezuela, and could have been introduced into the country with slaves from West Africa. The almost complete genome sequences of two strains confirmed its classification as a distinct subtype within G2. This study represents an in-depth analysis of the subtype diversity of HCV in Venezuela. HCV subtype diversity is still unexplored in the Americas and deserves further studies.

## Materials and Methods

This study was approved by the Bioethical Committee of Instituto Venezolano de Investigaciones Cientificas (IVIC). Serum samples were collected after written informed consent of the patients.

### PCR and sequencing

HCV genotype was determined by direct sequencing (Macrogen Service Center, Seoul, Korea) of a PCR-amplified product from the NS5B region. In order to maximize the probability of amplifying the NS5b region, the NS5b amplicon was generated by either one of three combinations of RT-nested PCR using: primers hep101-hep102 and primers hep101-hep105 in the first and second round [53], primers 8245P-8645N and 8276P-8580N in the first and second round [54] or a combination of these primers 8245P-hep102 and 8245P-8645N in the first and second round. Genotype was also previously assigned to these samples by sequencing the PCR product of the 5′NC region [11]. Core region was amplified by RT-nested PCR with primers 939P-C4N for the first round and combinations of C5P with C4N, C6N or C9N, in order to maximize the success in amplification (Table 1). Both sense and antisense inner primers were used for sequencing. Degenerate primers were designed to amplify the complete genome of G2j isolates, in regions exhibiting either conservation among G2 isolates. A total of 10 PCR overlapping fragments were sequenced, using primers described in Table 1:

- Fragment 1: 939P-C4N (1275nt), sequenced with primers 939P, C4N, C5P and 693P.
- Fragment 2: first round 939P-2001N, and second round either 693P-2001N for strain C1799 and 1298P-2001N for C1298, sequenced with primers 693P, 1298P, C4N, 2001N.
- Fragment 3: first round 2001P-3765N, and second round 2001P-3462N, sequenced with primers 2001P, 2451N, 2533P, 2533N, 3008N, 3105P, 3462N.
- Fragment 4: first round 2001P-5082N, and second round 3008P-5082N, sequenced with primers 3008P, 3185P, 4175P, 4175N, 4290P, 4290N, 3462N, 5082N.
- Fragment 5: first round 2001P-5930N, and second round 4175P-5309N, sequenced with primers 4175P, 5045P, 5045N, 5082P, 5082N, 5309N.
- Fragment 6: first round 2001P-6623N, and second round 5045P-6623N, sequenced with primers 5045P, 5930P, 5930N, 6100P, 6100N, 6522N, 6623N.
- Fragment 7: 5930P-7071N, sequenced with sense and antisense primers.
- Fragment 8: only for isolate C1799, first round 5930P-8645N, and second round 6712P-8625N, sequenced with primers 6712P, 7071P, 7071N, 7743P, PR3P, 8625N).
- Fragment 9: first round 5082P-102N, and second round 5930P-8645N, sequenced with primers 5930P, 6522P, 7071P, 7071N, 7670P, 7743P, 8245N, 8645N, for C1292 isolate. – Fragment 10: first round 7743P-9325N, and second round 8245P-9325N for C1799 and PR3P-9325N for C1292, both sequenced with primers 8245P, 8276P, 8645N, 9325N.

### Phylogenetic analysis

Sequence alignment was performed by the global alignment algorithm, using DNAman 5.2.2 (Lynnon Bio Soft, Canada). Phylogenetic analysis was performed either by the Neighbor Joining method (1000 bootstrap replicas, with genetic distances evaluated with Kimura 2 parameters corrections) (DNAman 5.2.2, Lynnon Bio Soft, Canada) or by Maximum Likelihood analysis, using the program PhyML version 3. Approximate Likelihood Ratio Test (aLRT) and the best-fit model analyzed here was selected with the Akaike Information Criterion (AIC) by using Modeltest Version 3.06 [55]: GTR model of substitution (4 Gamma rate categories). Newick trees application from Mega program was used for tree representation.

### Recombination analysis

Complete genome sequences were aligned and tested for possible recombination events involving sequences used in this study. We used two approaches implemented in the SimPlot program [56]: 1) a sliding window analysis of distances and 2) the bootscanning [57]. The SimPlot program was also used for establishing the similarity indexes along the HCV polyprotein.

### Substitution rate analysis

The substitution rate along the HCV polyprotein was measured using a sliding window by the procedure of Alvarez-Valin et al. [58]. Pairwise nucleotide distances (synonymous and non synonymous) within each window were estimated by the method of Comeron [59], as implemented in the computer program *k*-estimator (version 6.1), where *k* is the number of nucleotide substitutions between sequences. For those windows where this method could not be applied, the Jukes-Cantor [60] method was used for correction for multiple hits. The window had a size of 150 codons and a movement of 30.

### NS5A 3D protein structure prediction

The crystallographic structure of the NS5A protein from HCV genotype 2j strains is currently unknown. In order to model a 3D structure, we employed the most approximate structure available. For these reasons, crystallography data from NS5A domain I (amino acids 33 to 202) of HCV genotype 1b [23] was imported from PDB (accession number 3fgg), using the PDB ProteinWorkshop 3.6 program [61].

### Analysis of divergence time

Divergence times were estimated using Bayesian MCMC (Markov Chain Monte Carlo) analysis implemented in the BEAST program (http://evolve.zoo.ox.ac.uk/beast/) [62]. For this analysis, five population dynamics models were used: Bayesian, constant, exponential, expansion and logistic, using Akaike's Information Criterion to determine the model that best fits the data. Both strict and relaxed molecular clocks were employed to explore the extent of rate variation in the data as well as the age of the most recent common ancestor (MRCA). In all cases, the GTR model of nucleotide substitution was used with chain lengths of 170 million with the extent of convergence assessed using the Tracer program (http://evolve.zoo.ox.ac.uk/beast/). This analysis was run on three different data sets of NS5b sequences: genotype 1a (n = 94, 256 nt, 8301–8556), genotype 1b (n = 90, 256 nt, 8301–8556), genotype 2j (n = 62, 315 nt, 8301–8600).

Statistical differences were evaluated by the chi square test with Yate's correction, according to a computerized Epi Info program, version 3.3.2 (Centers for Disease Control and Prevention, Atlanta, GA, USA). The GenBank/EMBL/DDBJ accession numbers of the sequences reported in this paper are HM777048-HM777450, GU054427, GU054422, GU054421, GU054389, and GU054385.
